# Historical reviews of the assessment of human cardiovascular function: interrogation and understanding of the control of skin blood flow

**DOI:** 10.1007/s00421-019-04246-y

**Published:** 2019-11-27

**Authors:** David. A. Low, Helen Jones, N. Tim Cable, Lacy M. Alexander, W. Larry Kenney

**Affiliations:** 1 Research Institute for Sport and Exercise Sciences, Liverpool John Moores University, Liverpool, L3 3AF UK; 2 School of Sport, Exercise and Rehabilitation Sciences, University of Birmingham, Liverpool, UK; 3 Noll Laboratory, Department of Kinesiology, The Pennsylvania State University, University Park, PA USA

**Keywords:** Skin blood flow, Thermoregulation, Laser Doppler

## Abstract

Several techniques exist for the determination of skin blood flow that have historically been used in the investigation of thermoregulatory control of skin blood flow, and more recently, in clinical assessments or as an index of global vascular function. Skin blood flow measurement techniques differ in their methodology and their strengths and limitations. To examine the historical development of techniques for assessing skin blood flow by describing the origin, basic principles, and important aspects of each procedure and to provide recommendations for best practise. Venous occlusion plethysmography was one of the earliest techniques to intermittently index a limb’s skin blood flow under conditions in which local muscle blood flow does not change. The introduction of laser Doppler flowmetry provided a method that continuously records an index of skin blood flow (red cell flux) (albeit from a relatively small skin area) that requires normalisation due to high site-to-site variability. The subsequent development of laser Doppler and laser speckle imaging techniques allows the mapping of skin blood flow from larger surface areas and the visualisation of capillary filling from the dermal plexus in two dimensions. The use of iontophoresis or intradermal microdialysis in conjunction with laser Doppler methods allows for the local delivery of pharmacological agents to interrogate the local and neural control of skin blood flow. The recent development of optical coherence tomography promises further advances in assessment of the skin circulation via three-dimensional imaging of the skin microvasculature for quantification of vessel diameter and vessel recruitment.

## Introduction

The skin circulation is an expansive and richly innervated vascular bed. The ability of the skin circulation to adjust, adapt to, and support whole-body function and performance is crucial for, amongst other roles, thermoregulation (via optimisation of heat balance) and cardiovascular integration (e.g., maintenance of blood pressure). The ability to describe and understand the skin blood flow response to significant and varied stressors has implications across many areas of applied physiological research as well as clinical science.

In response to increased heat production via endogenous (e.g., exercise) or exogenous (e.g., high ambient temperatures) factors, large elevations in skin blood flow facilitate heat loss and thus the defence of internal temperature within physiological limits. Conversely, cold exposure typically requires reductions in skin blood flow approaching zero to limit heat loss. The large vascular network of the skin circulation and the sizeable range of potential changes in skin blood flow, ranging from near zero with severe cold stress to as high as 5–7 l/min with severe supine passive heating (Rowell 1974; Minson et al. 1998), means that the skin circulation is also an important site for the manipulation of vascular resistance for the modulation of blood pressure. More recently, the assessment of skin blood flow has been used as an index or predictor of global (cardio)vascular health. Dysfunction of the skin circulation is postulated to precede larger vessel impairment (Holowatz et al. 2008) and altered integrity of the skin circulation can infer the presence of subclinical cardiovascular disease (Hellmann et al. 2015). The ability to access and accurately measure skin blood flow is therefore of paramount importance for the understanding of human physiology and pathophysiology.

The skin is a readily accessible vascular bed and a variety of techniques have been developed over the years to provide relatively simple and non-invasive methods to assess skin microvascular function. The earliest recordings of human skin blood flow were made in the mid 1900s via whole-body calorimetry or helium exchange which led to estimates for whole-body skin blood flow under resting, normothermic conditions (Johnson et al. 2014). These techniques are quite challenging to use in more dynamic situations, e.g., environmental challenges or exercise, and, consequently, were not regularly used thereafter. Subsequent advances in the measurement of skin blood flow included water displacement or strain gauge plethysmography of a limb, followed by laser Doppler flowmetry with or without intradermal microdialysis. More recent and advanced methods involve laser Doppler imagery and optical coherence tomography. The aim of this review is to examine the historical development of measurement techniques for assessing skin blood flow. Skin microvasculature anatomy and control of skin blood flow will initially be covered. Thereafter, each measurement tool will be briefly described, including its origin and basic principles, important aspects of each procedure, and its strength and limitations. Finally, applications for the understanding of skin blood flow control and recommendations for promoting best practises in the current research will also be included.

## Skin circulation anatomy

The skin contains a highly specialised vascular network that is organised into two plexuses within the dermis that run parallel to the surface of the skin, located in the superficial and deep layers, respectively (Johnson et al. 2014). The majority of vessels, consisting of high-resistance terminal arterioles, papillary loops (true capillaries), and post-capillary venules, are located in the superficial papillary dermis, 1–2 mm beneath the epidermal surface. The papillary loops are a major determinant of heat exchange with the environment, being located in close proximity to the dermal–epidermal junction where there is both a high thermal gradient (due to the large surface area) and high blood flow (Johnson et al. 2014; Charkoudian and Stachenfeld 2014). Highly innervated arterioles control blood flow through the papillary loops, comprising an inner lining of endothelial cells encircled by a dual layer of vascular smooth muscle cells. A second vascular plexus is located at the dermal–subdermal junction, where the vessels are typically of greater diameter than those of the upper plexus, with 4–5 layers of vascular smooth muscle (Johnson et al. 2014). From this lower plexus, ascending arterioles connect to the upper plexus, hair follicles, and sweat glands. Despite the plexus and papillary loop arrangement being generically consistent, anatomical differences exist between skin regions. In the glabrous (non-hairy) skin of the palms, lips and plantar aspect of the feet, arteriovenous anastomoses (AVAs) bypass the resistance vessels, directly connecting the arterioles and venules (Johnson et al. 2014). As AVAs have a smaller surface area and lie deeper in the dermis than papillary loops, they are considered less efficient for thermoregulation, especially under certain conditions, e.g., upright exercise, manual labour, etc. (Johnson et al. 2014), but under resting conditions, glabrous skin of the hands and feet can significantly affect heat dissipation and conservation; for example, cold-induced vasodilation (Taylor et al. 2014).

## Skin blood flow control mechanisms

Precise control of blood flow to the skin is critical to thermoregulation and many aspects of cutaneous vascular control are unique to humans. At rest in thermoneutral environments, blood flow to the skin averages about 250 ml/min and comprises approximately 5% of cardiac output. Skin arterioles are richly innervated by efferent sympathetic neurons that, coupled with a variety of downstream signalling processes, allow the precise control of skin blood flow. For a more in-depth description of all the reflex neural and local mechanisms that underpin thermoregulatory control of skin blood flow, the reader is directed to a more detailed review (Johnson et al. 2014).

### Mechanisms of skin vasodilation

#### Reflex vasodilation

When core and/or mean skin temperatures are elevated, reflex skin vasodilation occurs in areas of skin not directly heated. Because the magnitude of increase in skin blood flow well exceeds that associated with passive withdrawal of constrictor tone, the mechanism underlying this response is termed *active vasodilation*. After central integration of core and/or skin temperatures, sympathetic nerve outflow increases to both eccrine sweat glands and skin arterioles. At the blood vessel level, skin sympathetic nerve terminals release acetylcholine (ACh) and additional as of yet not fully identified, co-transmitters (Kellogg et al. 1995). In a classical remaining mystery in physiology, putative neurotransmitter candidates include the neuropeptides vasoactive intestinal peptide (Wilkins et al. 2005; Bennett et al. 2003; Kellogg et al. 2007), substance P (Bogorad et al. 2015; Wong and Minson 2006, 2011; Wong et al. 2005), and calcitonin gene-related peptide (CGRP) (Savage et al. 1990; Schulze et al. 1997; Wallengren 1997; Wallengren et al. 1987; Wong and Minson 2011). Histamine receptor 1 (Wong et al. 2004) and neurokinin 1 receptor activation (Wong and Minson 2006) have also all been shown to contribute to active vasodilation (Holowatz et al. 2007).

Once stimulated, full expression of active skin vasodilation involves multiple downstream control mechanisms involving both endothelial cells and vascular smooth muscle cells. In addition to ACh- and co-transmitter-mediated effects on endothelial cells, histamine also increases skin blood flow, primarily through prostaglandin-mediated pathways, and a role for P2Y12 receptors possibly acting though platelets has also been proposed (Holowatz et al. 2010). Importantly, full expression of skin vasodilation also depends on the bioavailability of the intracellular mediator, nitric oxide (NO). NO is formed from the substrate l-arginine via endothelial nitric oxide synthase (eNOS)-catalysed mechanisms [ACh-mediated NO production (Shibasaki et al. 2002)] or direct stimulation from the putative neurotransmitters involved in active vasodilation (Bennett et al. 2003; Kellogg et al. 1998, 2007, 2008a, b; Zhao et al. 2004) and diffuses into vascular smooth muscle cells where it acts through soluble guanylate cyclase to decrease intracellular calcium concentration, leading to vasodilation of the vessel. Cyclooxygenase (COX)-dependent second messenger systems also contribute to active vasodilation (McCord et al. 2006). Finally, both inward rectifying potassium channels and ATP sensitive potassium channels induce vasodilation through hyperpolarisation of the vascular smooth muscle (Brunt et al. 2013).

#### Local heating

Local heating of the skin to temperatures below the pain threshold (~ 43 °C) produces a temperature-dependent increase in skin blood flow in the heated area. This response is mechanistically different from the reflex mechanisms that increase skin blood flow in response to increased body temperature and is characterised by a distinct pattern of the skin blood flow response. The pattern can be partitioned into two distinct biphasic increases in skin blood flow mediated by two independent mechanisms (Minson et al. 2001; Kellogg et al. 1999). The initial rapid increase in skin blood flow is mediated by a sensory axon reflex. After a brief nadir, the secondary phase consists of a more slowly developing rise to a stable plateau in skin blood flow that is predominantly NO-dependent.

The initial axon reflex phase of the local heating response is thought to be mediated by temperature-induced activation of C-fiber afferent neurons that release substance P and CGRP (Wong and Minson 2011). Additionally, neuropeptide Y and input from adrenergic nerves also modulate the temperature threshold at which the axon reflex occurs (Houghton et al. 2006; Hodges et al. 2008). NO contributes only modestly to the initial rise in skin blood flow with local heating but mediates approximately 70% of the secondary prolonged plateau phase of the local heating response. The NO-independent portion of the local heating plateau has been attributed to the eicosatrienoic acid family of endothelial-derived hyperpolarising factors (EDHF) (Brunt and Minson 2012).

### Mechanisms of skin vasoconstriction

#### Reflex vasoconstriction

Skin vasoconstriction (VC) is the initial thermoregulatory response to cold exposure, minimising convective and conductive heat loss to the environment through distinct reflex and local pathways that work both independently and co-operatively to maximise VC. Whole-body cold stress (i.e., decreased mean skin and/or core temperature) elicits reflex increases in efferent skin sympathetic nerve activity to skin sympathetic axon terminals, stimulating the release of neurotransmitters and co-transmitters from perivascular nerves that subserve skin vasoconstriction and subsequent reductions in skin blood flow (Charkoudian 2010; Greaney et al. 2014). While the VC response to whole-body cooling is entirely dependent on the sympathetic release of transmitters, only 60% of VC is mediated by noradrenaline, implicating the participation of sympathetic co-transmitter(s) in skin reflex VC to cold, including, possibly neuropeptide Y and ATP (Lang et al. 2017; Stephens et al. 2001, 2002, 2004; Lundberg 1996).

#### Local cooling

In contrast to reflex VC that is elicited by whole-body cooling, localised cooling of the skin blood vessels and surrounding tissue engages local VC mechanisms independent of efferent sympathetic reflex activity (Ekenvall et al. 1988; Pergola et al. 1993). Local (i.e., non-reflex) cold-induced VC is mediated primarily by noradrenaline at alpha-2-adrenoceptors (Cankar et al. 2004; Ekenvall et al. 1988; Johnson et al. 2005; Pergola et al. 1993) and by Rho kinase (Thompson-Torgerson et al. 2007), along with a proposed passive constriction via NO withdrawal (Hodges et al. 2006). Furthermore, the Rho/ROCK (Rho-associated protein kinase) and eNOS pathways are mutually inhibitory, whereby cGMP-dependent protein kinase inhibits Rho activation and ROCK, while Rho and ROCK downregulate eNOS expression and activity (Noma et al. 2006; Somlyo 2007).

Although the mechanisms of the function of these discrete control systems of skin blood flow have been generally well characterised, they do not only act in isolation, but, rather, they can be activated simultaneously under combinations of various stimuli. For example, during whole-body heat stress when neurally mediated active vasodilation is engaged, if a vasoconstrictor stimuli occurs, e.g., baroreflex activation, sympathetically neurally mediated vasoconstriction can be induced, albeit to a lesser degree due to a sympatholytic effect (Shibasaki et al. 2006). Similarly, locally mediated vasoconstriction can also be induced under whole-body heat stress conditions (Pergola et al. 1993) and, conversely, locally mediated vasodilation can also occur when superimposed on whole-body cold stress conditions when neurally mediated sympathetic vasoconstriction is engaged.

### Early methods of assessment

**Fig. 1:**
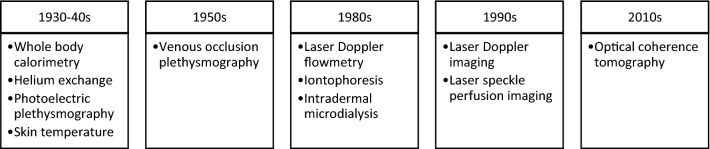
Timeline for the use of different methods for skin blood flow assessment in humans after the initial techniques of whole-body calorimetry, helium exchange, photoelectric plethysmography, and skin temperature

Initial studies of skin blood flow used whole-body calorimetry (Hardy and Soderstrom 1938) or the rate of absorption of helium through the skin (Behnke and Willmon 1940) to estimate whole-body skin blood flow in resting, normothermic conditions (see Fig. 1). Regional photoelectric plethysmography was also used to estimate regional and whole-body skin blood flows (Hertzman 1948). Photoelectric plethysmography detects blood volume changes in the microvasculature via recording changes in reflected light that has been injected into the skin through light-emitting diodes (Meglinski 2015). These techniques are quite challenging to conduct in dynamic conditions such as whole-body heating, so were not frequently used thereafter. Other early studies of skin blood flow relied on monitoring skin surface temperature changes based on the assumption that skin surface temperature follows changes in underlying skin blood flow (Grant and Holling 1938; Lewis and Pickering 1931). The rationale for this approach is that the temperature of the skin is directly affected by its rate of blood flow and heat conduction and convection to the body surface. Since the measurement of skin temperature is influenced by air temperature, it does not provide a quantitative measure of skin blood flow, and its relation to skin blood flow is nonlinear (Cooper and Cross 1949). That said, a fall in skin temperature or superficial shell (skin plus subcutaneous fat) insulation during heat exposure was used as an indicator of a neurally mediated increase in skin blood flow and the attendant vasodilation threshold. As a solution to the nonlinearity of the skin temperature:blood flow relationship, the assessment of the heat flow in regional skin areas using small surface calorimeters has been assessed (Fox et al. 1962).

**Fig. 2:**
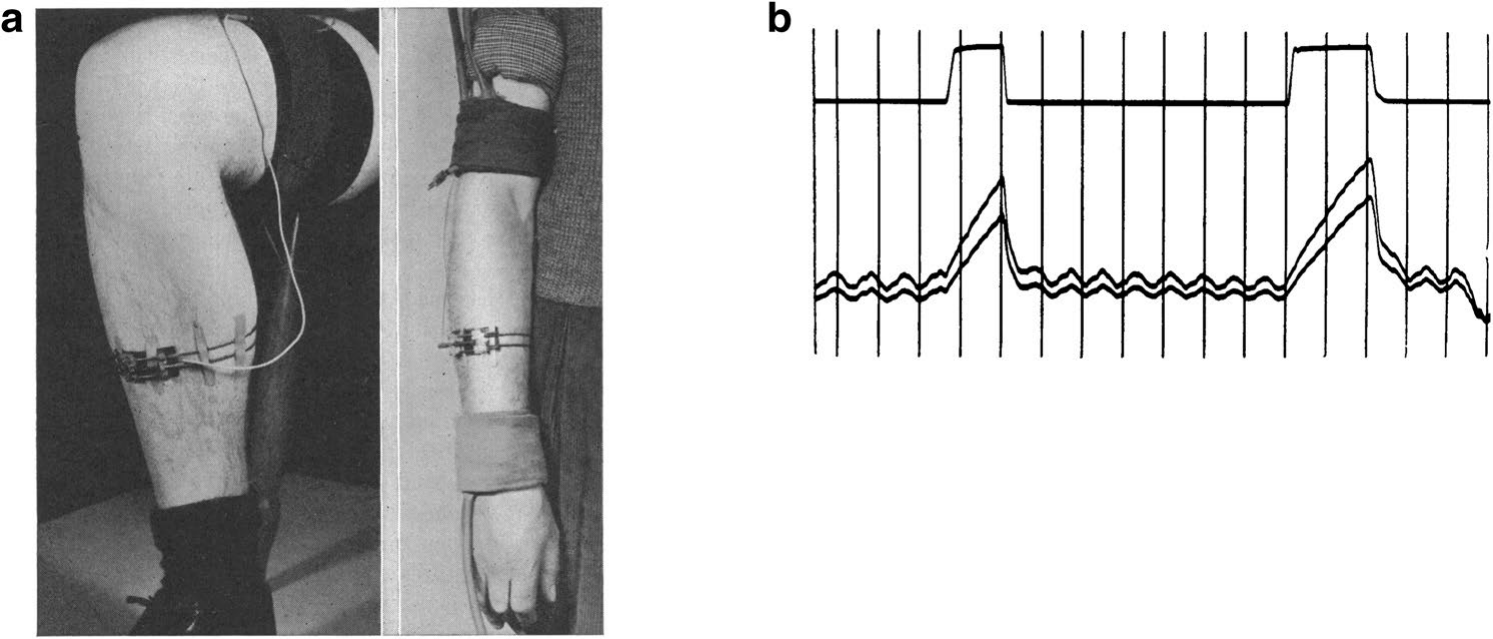
**a** Method of mounting a mercury strain gauge on a forearm and a calf. **b** Lower trace shows representative venous occlusion plethysmography records obtained with two forearm strain gauges with venous occlusion applied to the upper arm (upper trace shows venous occlusion pressure). Each interval is 4 s. From Whitney (1953) with permission

Subsequent studies detailing human skin blood flow responses to whole-body or local limb heat stress used venous occlusion plethysmography, most often with a mercury-in-silastic strain gauge as described by Whitney (see Fig. 2) (Whitney 1949, 1953). [Readers are referred to the recent article on methods for the determination of muscle blood flow for a more in-depth description and critique of this technique (Gliemann et al. 2018).] Briefly, a cuff placed around the proximal portion of a limb is inflated to a pressure greater than venous pressure but less than arterial pressure, so that venous volume (and limb circumference) increases at the rate of arterial inflow into the limb. Measurement of limb volume change via recordings of water displacement or circumference change from the strain gauge is then used to calculate the blood flow to the extremity in units of percent change per unit of time. The forearm has typically been used to assess the skin blood flow responses to various perturbations, such as thermal stress and leg exercise, but calf, hand, and foot blood flow can also be examined. For the measurement of calf or forearm blood flow, blood flow to the hand or foot is typically eliminated through an occlusive cuff inflated to suprasystolic levels, to remove the ambiguity between contributions from the hand or foot versus those from the proximal limb blood flow. Early studies of skin vascular responses reported elevations in forearm blood flow with whole-body heating (Cooper et al. 1954). Because the venous occlusion plethysmography method measures changes in total limb circumference, its utility in measuring changes in limb skin blood flow is based on evidence that inactive limb muscle blood flow does not increase during passive heating (Detry et al. 1972) or leg cycle exercise (Johnson and Rowell 1975). In these research approaches, the majority of the increase in blood flow to the limb is directed to the skin, but recent research using more modern technology has indicated that muscle blood flow can increase during local heating (Keller et al. 2010; Heinonen et al. 2011; Pearson et al. 2011; Chiesa et al. 2015).

Subsequent studies then demonstrated that skin blood flow was under sympathetic active vasodilator control by, either, arresting the elevation in skin blood flow in one forearm through adrenaline iontophoresis to the entire surface of that arm or rapidly decreasing skin blood flow using local nerve blocking agents during whole-body hyperthermia (Edholm et al. 1956, 1957). Later studies then discovered the biphasic skin blood flow response to local heating by examining the increase in skin blood flow with venous occlusion plethysmography while conducting whole arm heating with a water spray device to increase forearm temperature (Carberry et al. 1992; Johnson et al. 1976, 1986; Taylor et al. 1984).

#### Strengths

The venous occlusion plethysmography technique is straightforward and reproducible (Roberts et al. 1986). It can isolate blood flow to a limb and assess skin blood flow responses to a local perturbation, e.g., local heating, or administration of a pharmacological agent, or reflex responses to a whole-body stimulus, e.g., lower limb heating and/or exercise. Units are quantitative, at least in terms of ml per 100 ml tissue per minute.

#### Limitations

This technique only provides an indirect and intermittent measurement of limb blood flow and does not distinguish between muscle and skin blood flow (Cooper et al. 1955). The participant’s limb needs to be still during recordings, e.g., it is not possible to assess the blood flow of an exercising limb, and the occlusion of the hand or foot causes ischemia, which limits the duration of the measurement period. Cuff inflation may limit arterial inflow, while venous pressure is increased (Gliemann et al. 2018).

### Laser Doppler flowmetry

**Fig. 3:**
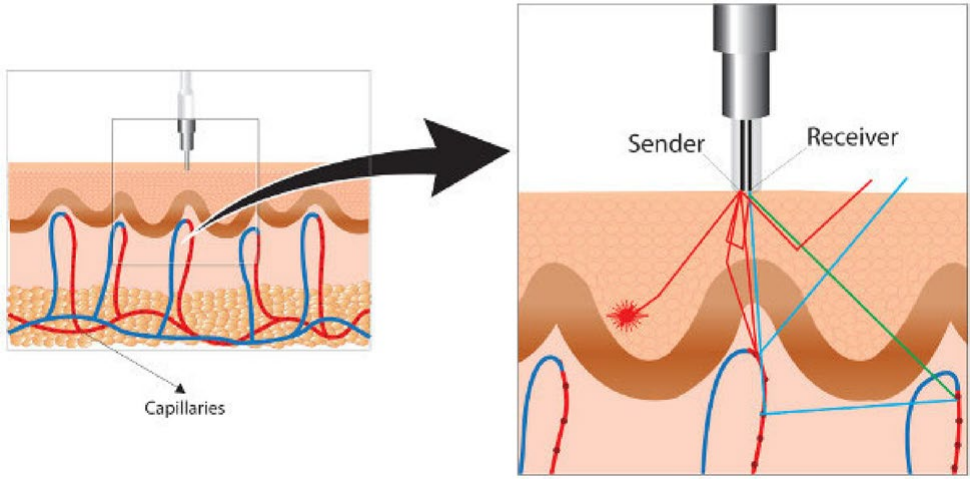
Laser Doppler assessment of skin blood flow where a beam of laser light is emitted from a fiber-optic probe (Sender). Light hitting moving blood cells undergoes a change in wavelength (Doppler shift), while light hitting static objects are unchanged. The magnitude and frequency distribution of these changes in wavelength are directly related to the number and velocity of the blood cells in the sample volume. The information is picked up by a returning fiber (receiver), converted into an electronic signal

The development and utilisation of laser Doppler flowmetry (LDF) has made it possible to reliably measure relative changes of an index of skin blood flow, independent of underlying muscle blood flow. LDF measures the Doppler shift induced by the movement of red blood cells through the skin vasculature (see Fig. 3; (Ahn et al. 1987; Briers 2001; Saumet et al. 1988). The output signal does not provide an absolute measure of flow, but is linearly related to blood flow. The first experimental derivation of this relationship was in the intestinal microvasculature of the cat (*r* = 0.96 between optical drop recording and laser Doppler) (Ahn et al. 1985).

Modern laser Doppler flowmeters assess red blood cell flux at a superficial depth and over a small volume of ~ 1 mm^3^ (see Table 1) with a high sampling frequency. Using laser Doppler flowmetry, skin blood flow to various local (e.g., local heating or cooling, cuff inflation/release, pharmacological administration as detailed below), or whole-body (e.g., dynamic or static exercise, whole-body heating/cooling) perturbations can be assessed. These data can provide important information on the neural or non-neural control of skin blood flow that may, in turn, provide an index of overall vascular health and function/dysfunction (Holowatz et al. 2008). Because of the small area and volume of skin under the laser Doppler flow probe and the inherent heterogeneity of the skin microvasculature, and direct site-to-site and day-to-day comparisons of the raw laser Doppler flux signal are highly variable (Braverman et al. 1990; Wardell et al. 1994; Dawson et al. 2015; Roberts et al. 2017). To account for potential anatomical differences, a normalisation process is typically utilised. Normalisation procedures of laser Doppler flux include converting the raw data to either (i) a percentage change from a physiological zero induced by arterial occlusion, (ii) a percentage change from a thermoneutral baseline (33°–34°), or a calculated percentage of (iii) a maximal vasodilation induced by either local heating (Johnson et al. 1984; Kellogg et al. 1993) or perfusion of an endothelium-independent vasodilator to directly act on the smooth muscle (e.g., sodium nitroprusside) (Minson et al. 2001; Kellogg et al. 1999; Abularrage et al. 2005; Rossi et al. 2002) or both. Normalisation to maximal vasodilation typically results in the least amount of variability between skin sites (Larsson et al. 2003; Roberts et al. 2017), but the most appropriate normalisation process should be experiment and hypothesis specific. For example, for studies examining skin blood flow responses to cold stress, e.g., reductions in skin blood flow, normalisation of the data to the pre-cold stress exposure, e.g., thermoneutral baseline, could be most appropriate (e.g., a % reduction or a delta change), but care should be taken with the potential for differences in baseline between groups and conditions. For investigations of skin blood flow responses to heat stress, e.g., increases in skin blood flow, normalisation of the data to maximal vasodilation could be optimal, but there are some important aspects to consider. Presenting skin blood flow data as a percentage of maximal vasodilation might conceal differences in absolute skin blood flow between different groups (e.g., a healthy vs. a diseased group) or different conditions (e.g., after a training intervention) that could change maximal vasodilation. This issue is important to consider when interpreting heat dissipation, because absolute (not relative or a percentage of maximal) skin blood flow influences skin temperature and, subsequently, the temperature gradient between the skin and the environment for heat exchange. Integrated multipoint laser Doppler probes that increase the volume of tissue under the probe reduce site-to-site variability (Roustit et al. 2010; Roberts et al. 2017). Similar to the use of venous occlusion plethysmography, the forearm or leg is most often used as the site of measurement of skin blood flow during experiments, but other skin sites are accessible as well, depending on the research question. One study assessing flux on the forearm, abdomen, thigh, and back saw no significant differences among sites with either whole-body heating or in response to infusion of graded concentrations of the neurotransmitter acetylcholine (Smith et al. 2013). Laser Doppler flowmetry is extremely sensitive to movement artefact and, therefore, use on an exercising limb is not feasible. A vacuum cushion around the experimental limb or other isolation method can be used to reduce movement artefact. Furthermore, in the majority of experimental settings examining the neurovascular control of skin blood flow, inferences about the vessel radius and thus vasomotor tone drive the research questions. Therefore, it is necessary to take driving pressure into account and normalise changes in skin blood flow (flux) to skin vascular conductance, i.e., divide by mean arterial blood pressure. From a practical standpoint, this requires an accurate measurement of arterial pressure changes and a correction for any potential orthostatic differential. A continuous or frequent measure of pressure is especially important during experimental perturbations where pressure may change, including static and dynamic exercise and whole-body heating and cooling.

**Table 1:** Ranges of sampling areas and penetrating depths of various techniques for assessing skin blood flow

Technique	Sampling area	Penetrating depth	Notes
Laser Doppler flowmetry	~ 1 mm^3^	0.3–0.5 mm	
Laser Doppler imaging	3 cm × 3 cm to 50 cm × 50 cm^a^	~ 0.5–1.5 mm^b^	^a^User defined ^b^Dependent on equipment wavelength and configuration
Laser Doppler speckle contrast imaging	5 mm × 7 mm to 24 cm × 24 cm^a^	150–300 µm^b^	^a^User defined ^b^Dependent on equipment wavelength and configuration
Optical coherence tomography	5 mm × 5 mm^a^	~ 300 μm^a^	^a^User defined

Typical sample frequencies for laser Doppler flowmetry are often ~ 32 Hz, which can be amplified and filtered for optimisation depending on the type of analysis. For example, at high sample frequencies, wavelet analysis can be performed to determine low-frequency periodic oscillations in flux measurements providing non-invasive mechanistic information on microvascular control mechanisms (Kastrup et al. 1989; Stefanovska et al. 1999). These periodic oscillations, or skin “flowmotion” (Bruning et al. 2015), represent the influence of heart beat (0.6–2.0 Hz), respiration (0.15–0.6 Hz), myogenic (~ 0.05 to 0.15 Hz) (Stefanovska et al. 1999), neurogenic (~ 0.02 to 0.05 Hz) (Kastrup et al. 1989; Soderstrom et al. 2003), and endothelial (~ 0.0095 to 0.02 Hz) influences on vascular smooth muscle relaxation (Kvandal et al. 2003, 2006; Rossi et al. 2008). Other potential applications for high-frequency data collection include determining neurovascular transduction with the simultaneous measurement of skin sympathetic nerve activity (Greaney and Kenney 2017). However, for the majority of applications, a sample frequency of 10–20 Hz is sufficient.

#### Strengths

Laser Doppler flowmetry is a simple technique that provides a continuous signal during various local or whole-body manoeuvres. With appropriate study design and data analyses, the technique is also reliable.

#### Limitations

Laser Doppler flowmetry only assesses skin blood flow over a small volume of skin and is extremely sensitive to movement artefact. Without appropriate normalisation, there can be large site-to-site and day-to-day heterogeneity. Measurement units are not quantitative and must be normalised based on the research question being asked.

### Coupling of laser Doppler flowmetry with other techniques

In the 1980s and 90s, iontophoresis and intradermal microdialysis techniques were developed that allowed the local application or perfusion of pharmacological agents into small regions of skin that could be combined with laser Doppler flowmetry or perfusion imaging (see below) for the detection of alterations in skin blood flow in response to the local delivery of the pharmacological substances to the skin. These developments allowed significant advances in the understanding of how skin blood flow is controlled.

### Iontophoresis

**Fig. 4:**
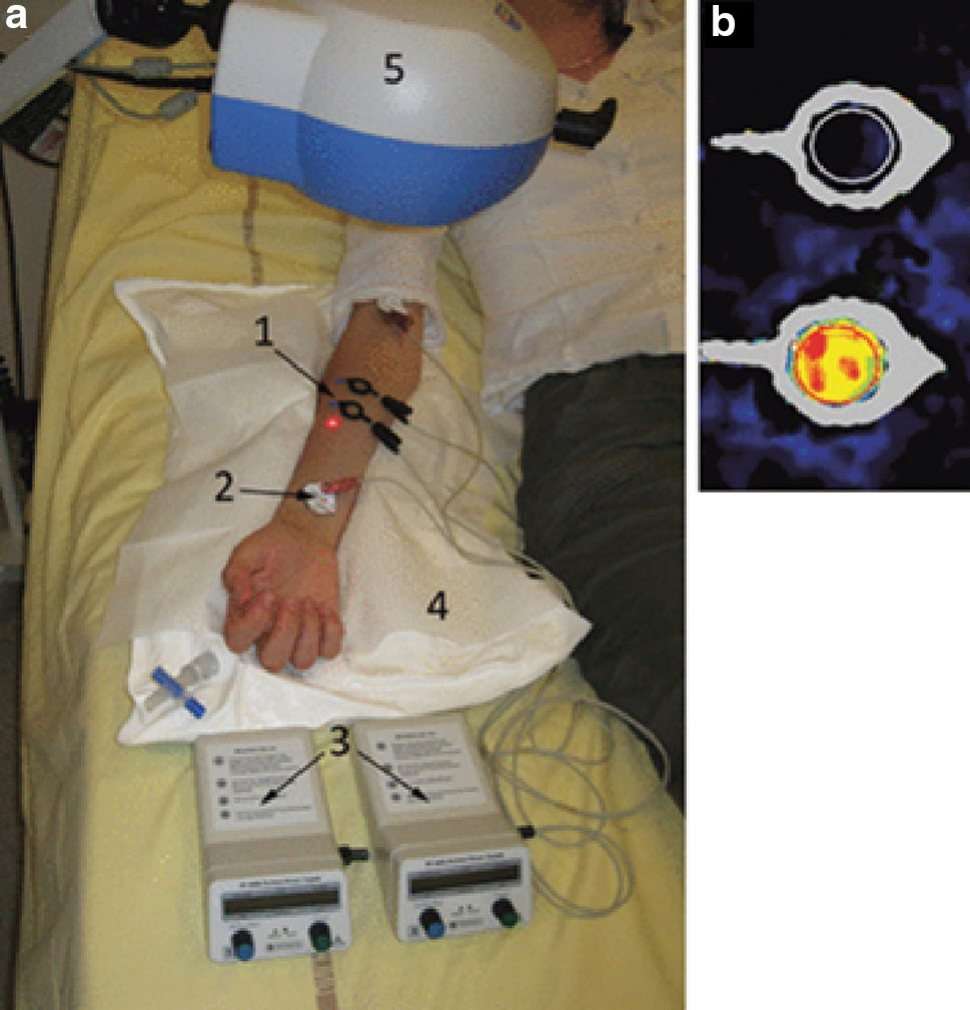
Example of an iontophoresis experimental setup. **a** Cathodal iontophoresis of a drug and control while recording skin blood flux with laser Doppler imaging (#5); (1) active electrode containing the drug, (2) passive electrode, (3) current generators connected to the electrodes, (4) vacuum cushion to reduce movement artefact. **b** Example of skin blood flux recorded during iontophoresis of sodium nitroprusside (bottom) and saline (top). With permission from Roustit and Cracowski (2012)

Iontophoresis is based on the principle that a charged drug in solution will migrate across the skin under the influence of a direct or alternating low-intensity electric current (see Fig. 4) (Kalia et al. 2004). The efficacy of the delivery of the compound of interest depends on a number of factors, including, the charge of the compound solution, the magnitude and duration of the current applied, the concentration and the pH of the solution, and the skin barrier (thickness, glabrous or non-glabrous, etc.).

The first studies that used iontophoresis for the investigation of skin blood flow applied noradrenaline, specific antagonists or alpha-1 or alpha-2 adrenergic agonists to a finger to characterise the adrenergic receptor subtypes in finger skin, showing that alpha-2 adrenoceptors were responsible for vasoconstriction induced by local cooling (Lindblad and Ekenvall 1986; Lindblad et al. 1986; Ekenvall et al. 1988). Soon after these studies, Kellogg and colleagues used bretylium iontophoresis, which blocks neurotransmitter release from adrenergic nerve terminals, in a small area of forearm skin to abolish adrenergic function and thus allow the examination of the active vasodilator system free of vasoconstrictor system activity (Kellogg et al. 1989, 1990, 1991, 1993). These studies significantly increased our understanding of the reflex control of active vasodilator activity by thermoregulatory reflexes as well as by other reflexes such as baroreflexes and those attending exercise (Kellogg et al. 1989, 1990, 1991, 1993). Thereafter, using local blockade of muscarinic receptors and cholinergic nerves Kellogg and colleagues also demonstrated for the first time that reflex vasodilation during whole-body heating was mediated through the release of sympathetic co-transmitter(s) (Kellogg et al. 1995). More recently, iontophoretic delivery of the endothelial dependent and independent vasodilators ACh and sodium nitroprusside has been used to assess endothelial function (Roustit and Cracowski 2013).

One of the issues with iontophoresis is nonspecific, current‐induced vasodilation that is a function of current density, charge, and duration (Tartas et al. 2005; Grossmann et al. 1995). Delaying the start of the experimental portion of a study for 45–60 min, so that blood flow returns to stable levels is possible, but this delay may allow the washout of the active drug, limiting the duration of efficacy of the agent in the experiment (Johnson et al. 2014). Topical anaesthesia before the iontophoresis application or including a control site may prevent or allow for quantitative correction of this issue (Cracowski and Roustit 2016). Furthermore, a nonpolar solvent as the only charge carrier without any competing ions should be used to deliver the active drug. Aqueous or saline solutions will mean that a substantial amount of the current will be carried by protons, hydroxide, sodium, or chloride ions that have greater electrical mobility than larger molecules and will therefore account for a large fraction of the current (Johnson et al. 2014). The use of deionized water as a vehicle limits the adjunction of competing ions, therefore enhancing iontophoretic transport but induces more current‐induced vasodilation (Cracowski and Roustit 2016).

### Strengths

Iontophoresis allows the simultaneous delivery of very small amounts of pharmacological compounds and monitoring of skin blood flow without any effect on the systemic circulation. The experimental setup is simple and easy to apply. Various pathways of the control of skin blood flow can be examined to further understand the mechanisms of skin blood flow control and/or skin vascular function in various populations.

#### Limitations

Unlike direct skin injection, the iontophoretic transport through the skin is not controlled and is, therefore, less precise in terms of drug delivery. The delivery of the drug can be affected by the medium and current‐induced vasodilation obfuscates the true response to the drug.

### Intradermal microdialysis

**Fig. 5:**
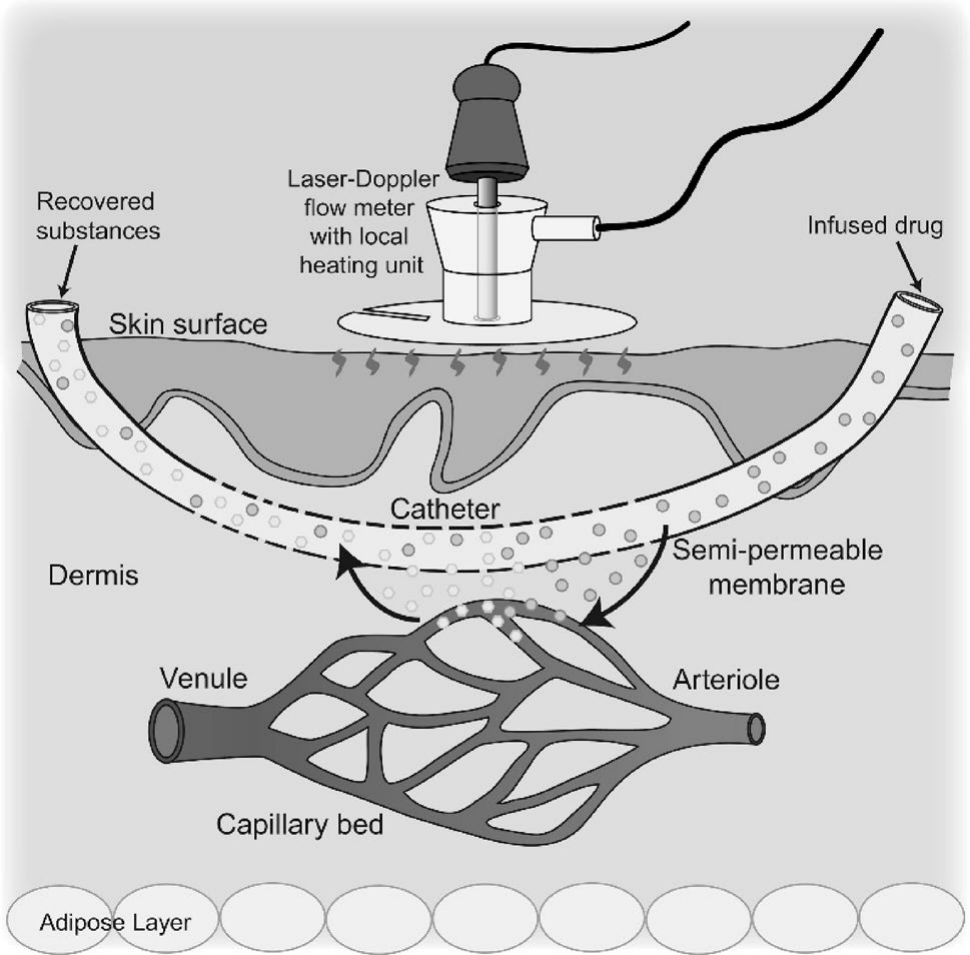
Schematic depiction of the intradermal microdialysis technique. A small dialysis membrane is inserted into the dermal skin layer, where it acts as an artificial capillary bed. Microdialysis allows pharmacological compounds to be delivered directly to an area of the skin vasculature without exerting systemic effects. Pairing of this technique with laser Doppler flowmetry allows for real-time measurement of skin blood flow changes during localised infusion of pharmacological substances. From Kenney (2017)

The use of microdialysis has the advantage of permitting delivery of higher molecular weight and/or nonpolar compounds that would be difficult to deliver by iontophoresis. The intradermal microdialysis technique was originally developed to sample neurotransmitters in neural tissue in rodent models (Ungerstedt and Hallstrom 1987). The technique has several applications ranging from the recovery of tissue metabolites (Petersen et al. 1992; Alba et al. 2018), delivery of pharmacological agents (Alexander et al. 2013; Craighead et al. 2017; Greaney et al. 2017; Smith et al. 2013, 2017), and estimation of localised blood flow in tissues (Hickner et al. 1992). Laser Doppler flux is the main outcome measure in studies utilising intradermal microdialysis (see Fig. 5) (Anderson et al. 1994). Microdialysis probes consist of a semi-permeable cellulose membrane and a hollow non-permeable guide tubing. Installation of the microdialysis probe is a minimally invasive procedure. Probes are placed in the tissue of interest utilising a fine gauge needle as an introducer. The microdialysis fiber is fed through the needle, which is then withdrawn and ~ 1 to 2 cm of the fiber is left in place in the dermis, with tubing connected at entry and exit ends of the fiber. A variety of different types of pore sizes are commercially available with 100 kDa and 30 kDa (CMA or BASi, respectively) being most consistently utilised in human skin experiments.

Insertion of the microdialysis probe stimulates an initial and transient vasodilatory response to the local tissue injury (Anderson et al. 1994) that requires a waiting period of an hour or more before beginning the experimental intervention. Furthermore, the presence of a probe may slightly increase the internal temperature threshold for active vasodilation and reduce the peak blood flow response to whole-body heating (Hodges et al. 2009). Temporarily anesthetising the skin with an ice pack prior to insertion of the probe abrogates this reduction in peak response to whole-body heating, but the increased threshold persists. A microdialysis probe inserted into a control (untreated) site that can be used as a comparative reference is, therefore, best practise.

Most studies of the neurovascular control of skin blood flow have utilised intradermal microdialysis for delivery of pharmacological agents, including agonists, antagonists, and cofactors to pharmacodissect the neurovascular signalling mechanisms underlying the control of skin blood flow. The delivery of agents via microdialysis is a function of its concentration and characteristics of the drug (hydro or lipophilic) (Groth 1996) in the perfusate, the flow rate of the perfusate (1–4 μL min^−1^), the molecular size (Clough 2005), and the pore size of the microdialysis membrane. The properties of the vehicle perfusate in which the drug is dissolved likewise need to be taken into consideration. Saline or Ringers solutions are typically used, but buffering or solubilising agents (ethanol or dimethyl sulfoxide) are often utilised to increase the molar concentration of the drug being perfused (Smith et al. 2017); thus, it is important to delineate any independent effects of these agents on neurovascular control mechanisms.

The initial studies that utilised intradermal microdialysis in the investigation of human skin blood flow provided the first data on the role of nitric oxide in neurally and locally mediated elevations in skin blood flow (Kellogg et al. 1998, 1999). Additionally, the use of intradermal microdialysis of bretylium tosylate, yohimbine, and propranolol (antagonists of pre-synaptic neurotransmitter release, ɑ-adrenoceptors, and β-adrenoceptors, respectively) built on previous systemic studies (Kenney et al. 1991, 1994) to verify the neurotransmitters contributing to skin reflex vasoconstriction. These studies revealed that while the vasoconstriction response to whole-body cooling is entirely dependent on the sympathetic release of transmitters, only 60% of vasoconstriction is mediated by noradrenaline (Stephens et al. 2001, 2004; Thompson-Torgerson et al. 2008).

#### Strengths

Pairing intradermal microdialysis with continuous measurement of skin blood flow has several advantages. This combined approach permits a strong repeated-measures within-subject experimental design. Each subject serves as his or her own control and in vivo experiments are performed in real time. Furthermore, this technology permits the study of the localised and immediate response of the skin microvasculature to thermal perturbation and drug perfusions without whole-body exposure or systemic drug effects (Debbabi et al. 2010; Holowatz et al. 2008). In addition, investigation of the efficacy of potential intervention strategies targeting a specific molecular signalling pathway can be explored before subjecting subjects to systemic interventions.

#### Limitations

The costs of the microdialysis probes can be prohibitive. The experimental setup is also technically challenging to ensure the optimal preparation and delivery of the various pharmacological substances. An extended period of time must ensue after needle insertion and to avoid the localised trauma affecting the subsequent skin blood flow assessment.

### New techniques to assess skin blood flow

Recent developments that can improve the spatial resolution over and above Laser Doppler flowmetry, which has been the most common and valid way to measure skin blood flow over the past 30 years, include optical techniques that image larger areas of skin. Over recent years, laser Doppler imaging and laser speckle perfusion imaging have been used to map the blood flow of the skin under investigation or visualise single capillary vessels within the skin in a two-dimensional format. Near infrared spectroscopy (NIRS) is another recently developed technology that assesses local tissue oxygenation and can provide indices of local oxygen consumption and blood flow (Ferrari and Quaresima 2012). NIRS is predominantly used for examining cerebral and skeletal muscle tissue hemodynamics due to the assumption that changes in the skin circulation do not modify the obtained data (Jones et al. 2016). Data suggest that if the light-source emitting and detector diodes are placed close together (< 20–25 mm), NIRS-derived measures of muscle oxygen saturation and blood volume are affected in conditions where skin blood flow is significantly altered, e.g., hyperthermia, and/or exercise (Tew et al. 2010). Readers are directed to previous reviews on NIRS for further information (Ferrari and Quaresima 2012; Jones et al. 2016).

### Laser Doppler imaging

**Fig. 6:**
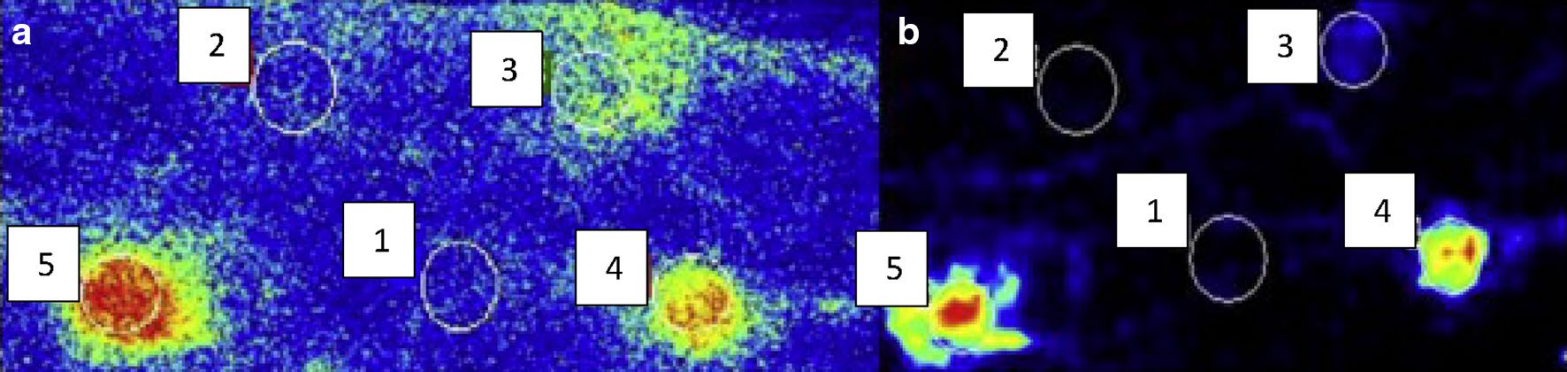
Examples of measurement of blood flux on different skin sites (numbered 1–5): unheated, heated to 36 °C, to 39 °C, to 42 °C and to 44 °C, respectively, using laser speckle contrast imaging (**a**) and laser Doppler imaging (**b**). From Millet et al. (2011) with permission

Laser Doppler imaging works on the same Doppler shift principle as laser Doppler flowmetry as described above. However, in laser Doppler imaging, four photodiodes and lenses are mounted in front of the laser and the beam passes in between the centre, on to a mirror which directs the beam to the target tissue (red blood cells). The scatter from the tissue is collected by the mirror back to the photodiodes. This method increases the diameter of the laser beam and allows a larger section of skin to be imaged (spatial resolution, see Fig. 6b). In using this technique, the equipment does not need to touch the skin and has been useful in assessing perfusion and depth of skin burns (Niazi et al. 1993). Nevertheless, the temporal resolution is limited by the time required to scan the beam as it may take up to 6 min to scan one small area of skin; thus, imaging techniques cannot be used to measure real-time continuous flow like single-site laser Doppler flowmetry.

#### Strengths

Laser Doppler imaging allows a larger section of skin to be interrogated and the equipment does not need to be in contact with the skin. Imaging also allows visual/qualitative analyses of responses alongside any objective data that might be simultaneously collected.

#### Limitations

It takes an extended period of time to scan an area of skin, so continuous real-time measurements of flow are not possible. The method does not provide quantitative indexes of blood flow in absolute flow units. The cost of the equipment is particularly prohibitive. The area of skin being assessed must remain still during recording.

### Laser speckle contrast imaging

Advancements in optical imaging technology to overcome temporal resolution issues have led to the emergence of laser speckle contrast imaging (LSCI). This technique provides a non-invasive, non-contact, continuous measurement of skin blood flow and works on the principle of tracking the speckle pattern, which is generated when tissue is illuminated by laser light. The speckle pattern changes when blood cells move within the region of interest (Briers et al. 2013). High levels of movement indicate an increase in velocity of the blood cells, producing a more blurred pattern that is associated with a reduction in contrast in that region. Low contrast, therefore, corresponds with high flow and high contrast corresponds with low flow (see Fig. 6a). The differences between the high and low contrasts are usually colour-coded within region of interest of the skin (e.g., the arm if measuring forearm blood flow). Whilst the technique of LSCI for obtaining measurements of blood flow was introduced in the 1980s (Fercher and Briers 1981), the first experiments on human skin were performed in 1996, with changes in speckle (low-contrast high flow and vice versa) evident following heating, cooling, rubbing of the skin, scalded skin with water (accidental), and in response to occlusion with a blood pressure cuff (Briers and Webster 1996). Since then the technique has been performed in a large number of research studies [e.g., (Tew et al. 2011)] and is increasingly used in clinical practise for dermatology to provide information on flow in conditions like Raynaud’s disease.

#### Strengths

LSCI produces real-time images using a high-frame rate (e.g. 25 Hz) which enables instantaneous tracking of blood flow changes with a high spatial and temporal resolution. This device is able to scan large skin surfaces (5 mm × 7 mm to 25 cm × 25 cm) to a depth of approximately 150–300 μm (see Table 1).

#### Limitations

LSCI does not provide direct visualisation to measure microvascular diameter which, therefore, does not provide quantitative indexes of blood flow in absolute flow units. The equipment may be prohibitively expensive. The area of skin being assessed must remain still during recording.

### Optical coherence tomography

Optical coherence tomography (OCT) is an immerging and novel three-dimensional imaging technique that is able to directly visualise the microvasculature to allow measurement of vessel diameter, speed and flow rate and visualise recruitment (density) of vessels in response to changing demands on the body. OCT can image to a greater depth (300 µm) but over smaller areas than laser speckle contrast imaging (see Table 1). The technique directs an optic beam (light) toward the tissue of interest and light that reflects back from the tissue is collected, while background noise is rejected. The first study to utilise this technique in the skin assessed skin burn scans to measure vascularity and found larger (hypertrophic) vessels in scar tissue from burns compared to normal skin (Liew et al. 2013). The analysis of the images was achieved using a speckle decorrelation algorithm (Liew et al. 2013). Similar, to laser perfusion imaging, the speckle pattern changes with movement of red blood cells compared to stationary tissue, which allows an estimate of the rate of blood flow. In recent studies from the same laboratory, OCT of the skin in the lower forearm was imaged alongside laser Doppler flowmetry during a 30 min bout of local heating to cause increases in skin blood flow (Carter et al. 2016, Smith et al. 2019). These studies uniquely demonstrated that the OCT technique can provide in-depth insight into the morphological and functional changes in skin vessels.

#### Strengths

Can image structural microvessels in the skin vasculature in three dimensions and to a greater depth than laser speckle contrast imaging while simultanously quantifying speed, flow rate and vessel recruitment.

#### Limitations

Does not provide information of absolute blood flow and cannot distinguish between vessels of different sizes (e.g., capillaries vs. small microvessels). Scanning takes time and currently possesses relatively poor temporal resolution. Current speckle decorrelation algorithms assess 2-D measures of vascularity.

## Summary/best practise

The choice of method for assessing skin blood flow depends on the interaction of a variety of factors such as the experimental design and the research/clinical aim, the participant characteristics, the available budget, and the expertise of the investigators. Here, we present our recommendations for adopting good technical practise to allow optimal collection and analyses of skin blood flow.

### Participant and site preparation

Depending on the exact research question or clinical assessment, investigators should ensure that participants start an experiment without any unwanted condition, which could transiently affect skin blood flow, for example, specific dietary intake, prior exercise or environmental exposure, and/or medication. Furthermore, the choice and condition of the site of interrogation should also be considered. Visible veins and particularly hairy regions should be avoided. Shaving of hair is sometimes conducted, but any shaving must be done at least 24 h prior to data collection due to the associated skin flare response. If resources and the experimental design allow consider using multiple integrated laser Doppler probes or imagers to increase the sampling area from which skin blood flow is indexed. Ensure that an appropriate amount of time is allowed after needle insertion for microdialysis membrane placement to avoid localised trauma affecting subsequent skin blood flow assessment. For repeated experimental trials, to reduce inter-trial site variation, use methods to assist with using the same site, e.g., photographs, anatomical landmarks, and/or temporary markings.

### Experimental setup

If possible, calibration of the chosen instrument should be conducted, e.g., using a calibration fluid for a known flux value for laser Doppler flowmetry, or electrical calibration for venous occlusion plethysmography. Ensure that the interrogated limb/body part is stationary and will remain static throughout data collection, which is often dependent on participant comfort. For delivery of pharmacological agents using microdialysis membranes, the perfusate, the flow rate of the perfusate, and the molecular size and pore size of the microdialysis membrane need to be considered and recorded.

### Data collection

Record arterial blood pressure using continuous digital photoplethysmography. If intermittent arterial blood pressure is to be recorded using automated or manual sphygmomanometry measure blood pressure from the opposite limb if arm or leg skin blood flow is being assessed to avoid the blood pressure measurement, e.g., arterial occlusion, interfering with the skin blood flow signal. The recording of local skin temperature at or near the skin blood flow assessment site is also beneficial. The requirements for the continuous collection of skin blood flow and the preferred size of the sampling area of skin blood flow should be considered, because laser Doppler probes allow continuous recordings, but laser Doppler imagers typically do not. When using iontophoresis, topical anaesthesia or a control site may prevent or allow for quantitative correction of current induced hyperemia.

### Data analyses

For laser Doppler flux data, normalise data to mean arterial blood pressure to calculate skin vascular conductance to take driving pressure into account. To avoid site-to-site and day-to-day variability laser in Doppler flux data normalise data as a percentage change from a physiological zero or a thermoneutral baseline or a calculated percentage of maximal skin vasodilation. For the examination of vasoconstrictor responses to sympathetic stimuli percentage or delta change data are typically used, but consider baseline absolute skin blood flow when interpreting differences between groups/conditions. When assessing vasodilatory responses to heating normalisation to maximal vasodilation is common, but analysis of the maximal vasodilation data as well as the absolute skin blood flow responses are preferred when comparing between groups or conditions. For completeness, an analyses and presentation of both absolute and relative skin blood flow data as well as local maximal vasodilation data are optimal.

### Perspectives

Several techniques have been developed over the years for the determination of skin blood flow and have allowed significant advances in the understanding of thermoregulatory control of skin blood flow in response to environmental stressors, and/or during various exercise stimuli in both healthy and diseased populations. Furthermore, the measurement of skin blood flow has been incorporated into clinical assessments and has been used as an index of global vascular function. The development of laser Doppler flowmetry provides a continuous index of skin blood flow (red cell flux) during various local or whole-body perturbations with the advent of laser Doppler imaging and laser speckle perfusion imaging techniques allowing a similar index of skin blood flow from larger skin surface areas albeit intermittently. Because of the ease of access to the skin surface, these measurements of skin blood flow are typically straightforward. Clear limitations are, however, site-to-site variability and the small regional sites of measurement. Advances that allow greater areas of measurement would benefit these techniques. Laser Doppler methods do allow the simultaneous use of iontophoresis or intradermal microdialysis for the local delivery of pharmacological agents to interrogate the local and neural control of skin blood flow. Such approaches, although technically challenging, can provide important information on the mechanisms of the control of skin blood flow. Laser Doppler techniques quantify the speed of red blood cell flux from the vast and intricate network of skin blood vessels rather than flow per se. Ideally, although challenging, methods that permit the quantification of skin blood flow from specific branches of skin microvessels would help to advance the methodology in this field and the understanding of the control of skin blood flow. The recent development of optical coherence tomography that allows three-dimensional imaging of the skin microvasculature for quantification of vessel diameter and recruitment of vessels promises further advances in the assessment of skin blood flow.

## Abbreviations

ACh: Acetylcholine
ATP: Adenosine triphosphate
AVAs: Arteriovenous anastomoses
CGRP: Calcitonin gene-related peptide
EDHF: Endothelial-derived hyperpolarising factors
eNOS: Endothelial nitric oxide synthase
LDF: Laser Doppler flowmetry
LSCI: Laser speckle contrast imaging
NO: Nitric oxide
OCT: Optical coherence tomography
ROCK: Rho-associated protein kinase
VC: Skin vasoconstriction

## References

[CR1] Abularrage CJ, Sidawy AN, Aidinian G, Singh N, Weiswasser JM, Arora S (2005). Evaluation of macrocirculatory endothelium-dependent and endothelium-independent vasoreactivity in vascular disease. Perspect Vasc Surg Endovasc Ther.

[CR2] Ahn H, Lindhagen J, Nilsson GE, Salerud EG, Jodal M, Lundgren O (1985). Evaluation of laser Doppler flowmetry in the assessment of intestinal blood flow in cat. Gastroenterology.

[CR3] Ahn H, Johansson K, Lundgren O, Nilsson GE (1987). In vivo evaluation of signal processors for laser Doppler tissue flowmeters. Med Biol Eng Comput.

[CR4] Alba BK, Greaney JL, Ferguson SB, Alexander LM (2018). Endothelial function is impaired in the cutaneous microcirculation of adults with psoriasis through reductions in nitric oxide-dependent vasodilation. Am J Physiol Heart Circ Physiol.

[CR5] Alexander LM, Kutz JL, Kenney WL (2013). Tetrahydrobiopterin increases NO-dependent vasodilation in hypercholesterolemic human skin through eNOS-coupling mechanisms. Am J Physiol Regul Integr Comp Physiol.

[CR6] Anderson C, Andersson T, Wardell K (1994). Changes in skin circulation after insertion of a microdialysis probe visualized by laser Doppler perfusion imaging. J Invest Dermatol.

[CR7] Behnke AR, Willmon TL (1940). Cutaneous diffusion of helium in relation to peripheral blood flow and the absorption of atmospheric nitrogen through the skin. Am J Physiol.

[CR8] Bennett LA, Johnson JM, Stephens DP, Saad AR, Kellogg DL (2003). Evidence for a role for vasoactive intestinal peptide in active vasodilatation in the cutaneous vasculature of humans. J Physiol.

[CR9] Bogorad MI, DeStefano J, Karlsson J, Wong AD, Gerecht S, Searson PC (2015). Review: in vitro microvessel models. Lab Chip.

[CR10] Braverman IM, Keh A, Goldminz D (1990). Correlation of laser Doppler wave patterns with underlying microvascular anatomy. J Invest Dermatol.

[CR11] Briers JD (2001). Laser Doppler, speckle and related techniques for blood perfusion mapping and imaging. Physiol Meas.

[CR12] Briers JD, Webster S (1996). Laser speckle contrast analysis (LASCA): a nonscanning, full-field technique for monitoring capillary blood flow. J Biomed Opt.

[CR13] Briers D, Duncan DD, Hirst E, Kirkpatrick SJ, Larsson M, Steenbergen W, Stromberg T, Thompson OB (2013). Laser speckle contrast imaging: theoretical and practical limitations. J Biomed Opt.

[CR14] Bruning RS, Kenney WL, Alexander LM (2015). Altered skin flowmotion in hypertensive humans. Microvasc Res.

[CR15] Brunt VE, Minson CT (2012). KCa channels and epoxyeicosatrienoic acids: major contributors to thermal hyperaemia in human skin. J Physiol.

[CR16] Brunt VE, Fujii N, Minson CT (2013). No independent, but an interactive, role of calcium-activated potassium channels in human cutaneous active vasodilation. J Appl Physiol.

[CR17] Cankar K, Finderle Z, Strucl M (2004). The role of alpha1- and alpha2-adrenoceptors in gender differences in cutaneous LD flux response to local cooling. Microvasc Res.

[CR18] Carberry PA, Shepherd AM, Johnson JM (1992). Resting and maximal forearm skin blood flows are reduced in hypertension. Hypertension.

[CR19] Carter HH, Gong P, Kirk RW, Es’haghian S, Atkinson CL, Sampson DD, Green DJ, McLaughlin RA (2016). Optical coherence tomography in the assessment of acute changes in cutaneous vascular diameter induced by heat stress. J Appl Physiol.

[CR20] Charkoudian N (2010). Mechanisms and modifiers of reflex induced cutaneous vasodilation and vasoconstriction in humans. J Appl Physiol.

[CR21] Charkoudian N, Stachenfeld NS (2014). Reproductive hormone influences on thermoregulation in women. Comp Physiol.

[CR22] Chiesa ST, Trangmar SJ, Kalsi KK, Rakobowchuk M, Banker DS, Lotlikar MD, Ali L, Gonzalez-Alonso J (2015). Local temperature-sensitive mechanisms are important mediators of limb tissue hyperemia in the heat-stressed human at rest and during small muscle mass exercise. Am J Physiol Heart Circ Physiol.

[CR23] Clough GF (2005). Microdialysis of large molecules. AAPS J.

[CR24] Cooper KE, Cross KW (1949). A comparison of methods for gauging the blood flow through the hand. Clin Sci (Lond).

[CR25] Cooper KE, Edholm OG, Fletcher JG, Fox RH, Macpherson RK (1954). Vasodilatation in the forearm during indirect heating. J Physiol.

[CR26] Cooper KE, Edholm OG, Mottram RF (1955). The blood flow in skin and muscle of the human forearm. J Physiol.

[CR27] Cracowski JL, Roustit M (2016). Current methods to assess human cutaneous blood flow: an updated focus on laser-based-techniques. Microcirculation.

[CR28] Craighead DH, McCartney NB, Tumlinson JH, Alexander LM (2017). Mechanisms and time course of menthol-induced cutaneous vasodilation. Microvasc Res.

[CR29] Dawson EA, Low DA, Meeuwis IH, Kerstens FG, Atkinson CL, Cable NT, Green DJ, Thijssen DH (2015). Reproducibility of cutaneous vascular conductance responses to slow local heating assessed using seven-laser array probes. Microcirculation.

[CR30] Debbabi H, Bonnin P, Ducluzeau PH, Leftheriotis G, Levy BI (2010). Noninvasive assessment of endothelial function in the skin microcirculation. Am J Hypertens.

[CR31] Detry JM, Brengelmann GL, Rowell LB, Wyss C (1972). Skin and muscle components of forearm blood flow in directly heated resting man. J Appl Physiol.

[CR32] Edholm OG, Fox RH, Macpherson RK (1956). The effect of body heating on the circulation in skin and muscle. J Physiol.

[CR33] Edholm OG, Fox RH, Macpherson RK (1957). Vasomotor control of the cutaneous blood vessels in the human forearm. J Physiol.

[CR34] Ekenvall L, Lindblad LE, Norbeck O, Etzell BM (1988). Alpha-adrenoceptors and cold-induced vasoconstriction in human finger skin. Am J Physiol.

[CR35] Fercher AF, Briers JD (1981). Flow visualization by means of single-exposure speckle photography. Opt Commun.

[CR36] Ferrari M, Quaresima V (2012). A brief review on the history of human functional near-infrared spectroscopy (fNIRS) development and fields of application. Neuroimage.

[CR37] Fox RH, Goldsmith R, Kidd DJ (1962). Cutaneous vasomotor control in the human head, neck and upper chest. J Physiol.

[CR38] Gliemann L, Mortensen SP, Hellsten Y (2018). Methods for the determination of skeletal muscle blood flow: development, strengths and limitations. Eur J Appl Physiol.

[CR39] Grant R, Holling H (1938). Further observations on the vascular responses of the human limb to body warming: evidence for sympathetic vasodilator nerves in the normal subject. Clin Sci (Lond).

[CR40] Greaney JL, Kenney WL (2017). Measuring and quantifying skin sympathetic nervous system activity in humans. J Neurophysiol.

[CR41] Greaney JL, Stanhewicz AE, Kenney WL, Alexander LM (2014). Muscle sympathetic nerve activity during cold stress and isometric exercise in healthy older adults. J Appl Physiol.

[CR42] Greaney JL, Kutz JL, Shank SW, Jandu S, Santhanam L, Alexander LM (2017). Impaired hydrogen sulfide-mediated vasodilation contributes to microvascular endothelial dysfunction in hypertensive adults. Hypertension.

[CR43] Grossmann M, Jamieson MJ, Kellogg DL, Kosiba WA, Pergola PE, Crandall CG, Shepherd AM (1995). The effect of iontophoresis on the cutaneous vasculature: evidence for current-induced hyperemia. Microvasc Res.

[CR44] Groth L (1996). Cutaneous microdialysis. Methodology and validation. Acta Derm Venereol Suppl (Stockh).

[CR45] Hardy JD, Soderstrom GF (1938). Heat loss from the nude body and peripheral blood flow at temperatures of 22 °C to 35 °C two figures. J Nutr.

[CR46] Heinonen I, Brothers RM, Kemppainen J, Knuuti J, Kalliokoski KK, Crandall CG (2011). Local heating, but not indirect whole body heating, increases human skeletal muscle blood flow. J Appl Physiol.

[CR47] Hellmann M, Roustit M, Cracowski JL (2015). Skin microvascular endothelial function as a biomarker in cardiovascular diseases?. Pharmacol Rep.

[CR48] Hertzman AB (1948). Photoelectric plethysmography of the skin. Methods Med Res.

[CR49] Hickner RC, Rosdahl H, Borg I, Ungerstedt U, Jorfeldt L, Henriksson J (1992). The ethanol technique of monitoring local blood flow changes in rat skeletal muscle: implications for microdialysis. Acta Physiol Scand.

[CR50] Hodges GJ, Zhao K, Kosiba WA, Johnson JM (2006). The involvement of nitric oxide in the cutaneous vasoconstrictor response to local cooling in humans. J Physiol.

[CR51] Hodges GJ, Kosiba WA, Zhao K, Johnson JM (2008). The involvement of norepinephrine, neuropeptide Y, and nitric oxide in the cutaneous vasodilator response to local heating in humans. J Appl Physiol.

[CR52] Hodges GJ, Chiu C, Kosiba WA, Zhao K, Johnson JM (2009). The effect of microdialysis needle trauma on cutaneous vascular responses in humans. J Appl Physiol.

[CR53] Holowatz LA, Thompson-Torgerson CS, Kenney WL (2007). Altered mechanisms of vasodilation in aged human skin. Exerc Sport Sci Rev.

[CR54] Holowatz LA, Thompson-Torgerson CS, Kenney WL (2008). The human cutaneous circulation as a model of generalized microvascular function. J Appl Physiol.

[CR55] Holowatz LA, Jennings JD, Lang JA, Kenney WL (2010). Systemic low-dose aspirin and clopidogrel independently attenuate reflex cutaneous vasodilation in middle aged humans. J Appl Physiol.

[CR56] Houghton BL, Meendering JR, Wong BJ, Minson CT (2006). Nitric oxide and noradrenaline contribute to the temperature threshold of the axon reflex response to gradual local heating in human skin. J Physiol.

[CR57] Johnson JM, Rowell LB (1975). Forearm skin and muscle vascular responses to prolonged leg exercise in man. J Appl Physiol.

[CR58] Johnson JM, Brengelmann GL, Rowell LB (1976). Interactions between local and reflex influences on human forearm skin blood flow. J Appl Physiol.

[CR59] Johnson JM, Taylor WF, Shepherd AP, Park MK (1984). Laser-Doppler measurement of skin blood flow: comparison with plethysmography. J Appl Physiol.

[CR60] Johnson JM, O’Leary DS, Taylor WF, Kosiba W (1986). Effect of local warming on forearm reactive hyperaemia. Clin Physiol.

[CR61] Johnson JM, Yen TC, Zhao K, Kosiba WA (2005). Sympathetic, sensory, and nonneuronal contributions to the cutaneous vasoconstrictor response to local cooling. Am J Physiol Heart Circ Physiol.

[CR62] Johnson JM, Minson CT, Kellogg DL (2014). Cutaneous vasodilator and vasoconstrictor mechanisms in temperature regulation. Compr Physiol.

[CR63] Jones S, Chiesa ST, Chaturvedi N, Hughes AD (2016). Recent developments in near-infrared spectroscopy (NIRS) for the assessment of local skeletal muscle microvascular function and capacity to utilise oxygen. Artery Res.

[CR64] Kalia YN, Naik A, Garrison J, Guy RH (2004). Iontophoretic drug delivery. Adv Drug Deliv Rev.

[CR65] Kastrup J, Bulow J, Lassen NA (1989). Vasomotion in human skin before and after local heating recorded with laser Doppler flowmetry. A method for induction of vasomotion. Int J Microcirc Clin Exp.

[CR66] Keller DM, Sander M, Stallknecht B, Crandall CG (2010). Alpha-adrenergic vasoconstrictor responsiveness is preserved in the heated human leg. J Physiol.

[CR67] Kellogg DL, Johnson JM, Kosiba WA (1989). Selective abolition of adrenergic vasoconstrictor responses in skin by local iontophoresis of bretylium. Am J Physiol Heart Circ Physiol.

[CR68] Kellogg DL, Johnson JM, Kosiba WA (1990). Baroreflex control of the cutaneous active vasodilator system in humans. Circ Res.

[CR69] Kellogg DL, Johnson JM, Kosiba WA (1991). Competition between cutaneous active vasoconstriction and active vasodilation during exercise in humans. Am J Physiol Heart Circ Physiol.

[CR70] Kellogg DL, Johnson JM, Kenney WL, Pergola PE, Kosiba WA (1993). Mechanisms of control of skin blood flow during prolonged exercise in humans. Am J Physiol.

[CR71] Kellogg DL, Pergola PE, Piest KL, Kosiba WA, Crandall CG, Grossmann M, Johnson JM (1995). Cutaneous active vasodilation in humans is mediated by cholinergic nerve cotransmission. Circ Res.

[CR72] Kellogg DL, Crandall CG, Liu Y, Charkoudian N, Johnson JM (1998). Nitric oxide and cutaneous active vasodilation during heat stress in humans. J Appl Physiol.

[CR73] Kellogg DL, Liu Y, Kosiba IF, O’Donnell D (1999). Role of nitric oxide in the vascular effects of local warming of the skin in humans. J Appl Physiol.

[CR74] Kellogg DL, Hodges GJ, Orozco CR, Phillips TM, Zhao JL, Johnson JM (2007). Cholinergic mechanisms of cutaneous active vasodilation during heat stress in cystic fibrosis. J Appl Physiol.

[CR75] Kellogg DL, Zhao JL, Wu Y (2008). Endothelial nitric oxide synthase control mechanisms in the cutaneous vasculature of humans in vivo. Am J Physiol Heart Circ Physiol.

[CR76] Kellogg DL, Zhao JL, Wu Y (2008). Neuronal nitric oxide synthase control mechanisms in the cutaneous vasculature of humans in vivo. J Physiol.

[CR77] Kenney WL (2017). Edward F. Adolph distinguished lecture: skin-deep insights into vascular aging. J Appl Physiol.

[CR78] Kenney WL, Tankersley CG, Newswanger DL, Puhl SM (1991). Alpha 1-adrenergic blockade does not alter control of skin blood flow during exercise. Am J Physiol.

[CR79] Kenney WL, Zappe DH, Tankersley CG, Derr JA (1994). Effect of systemic yohimbine on the control of skin blood flow during local heating and dynamic exercise. Am J Physiol.

[CR80] Kvandal P, Stefanovska A, Veber M, Kvernmo HD, Kirkeboen KA (2003). Regulation of human cutaneous circulation evaluated by laser Doppler flowmetry, iontophoresis, and spectral analysis: importance of nitric oxide and prostaglandines. Microvasc Res.

[CR81] Kvandal P, Landsverk SA, Bernjak A, Stefanovska A, Kvernmo HD, Kirkeboen KA (2006). Low-frequency oscillations of the laser Doppler perfusion signal in human skin. Microvasc Res.

[CR82] Lang JA, Krajek AC, Smaller KA (2017). Evidence for a functional vasoconstrictor role for ATP in the human cutaneous microvasculature. Exp Physiol.

[CR83] Larsson M, Nilsson H, Stromberg T (2003). In vivo determination of local skin optical properties and photon path length by use of spatially resolved diffuse reflectance with applications in laser Doppler flowmetry. Appl Opt.

[CR84] Lewis T, Pickering GW (1931). Vasodilation in the limbs in response to warming the body; with evidence for sympathetic vasodilator nerves in man. Heart.

[CR85] Liew YM, McLaughlin RA, Gong P, Wood FM, Sampson DD (2013). In vivo assessment of human burn scars through automated quantification of vascularity using optical coherence tomography. J Biomed Opt.

[CR86] Lindblad LE, Ekenvall L (1986). Alpha-adrenoceptors in the vessels of human finger skin. Acta Physiol Scand.

[CR87] Lindblad LE, Ekenvall L, Ancker K, Rohman H, Oberg PA (1986). Laser Doppler flow-meter assessment of iontophoretically applied norepinephrine on human finger skin circulation. J Invest Dermatol.

[CR88] Lundberg JM (1996). Pharmacology of cotransmission in the autonomic nervous system: integrative aspects on amines, neuropeptides, adenosine triphosphate, amino acids and nitric oxide. Pharmacol Rev.

[CR89] McCord GR, Cracowski JL, Minson CT (2006). Prostanoids contribute to cutaneous active vasodilation in humans. Am J Physiol Regul Integr Comp Physiol.

[CR90] Meglinski I (2015). Biophotonics for medical applications.

[CR91] Millet C, Roustit M, Blaise S, Cracowski JL (2011). Comparison between laser speckle contrast imaging and laser Doppler imaging to assess skin blood flow in humans. Microvasc Res.

[CR92] Minson CT, Wladkowski SL, Cardell AF, Pawelczyk JA, Kenney WL (1998). Age alters the cardiovascular response to direct passive heating. J Appl Physiol.

[CR93] Minson CT, Berry LT, Joyner MJ (2001). Nitric oxide and neurally mediated regulation of skin blood flow during local heating. J Appl Physiol.

[CR94] Niazi ZB, Essex TJ, Papini R, Scott D, McLean NR, Black MJ (1993). New laser Doppler scanner, a valuable adjunct in burn depth assessment. Burns.

[CR95] Noma K, Oyama N, Liao JK (2006). Physiological role of ROCKs in the cardiovascular system. Am J Physiol Cell Physiol.

[CR96] Pearson J, Low DA, Stohr E, Kalsi K, Ali L, Barker H, Gonzalez-Alonso J (2011). Hemodynamic responses to heat stress in the resting and exercising human leg: insight into the effect of temperature on skeletal muscle blood flow. Am J Physiol Regul Integr Comp Physiol.

[CR97] Pergola PE, Kellogg DL, Johnson JM, Kosiba WA, Solomon DE (1993). Role of sympathetic nerves in the vascular effects of local temperature in human forearm skin. Am J Physiol.

[CR98] Petersen LJ, Kristensen JK, Bulow J (1992). Microdialysis of the interstitial water space in human skin in vivo: quantitative measurement of cutaneous glucose concentrations. J Invest Dermatol.

[CR99] Roberts DH, Tsao Y, Breckenridge AM (1986). The reproducibility of limb blood flow measurements in human volunteers at rest and after exercise by using mercury-in-Silastic strain gauge plethysmography under standardized conditions. Clin Sci (Lond).

[CR100] Roberts KA, van Gent T, Hopkins ND, Jones H, Dawson EA, Draijer R, Carter HH, Atkinson CL, Green DJ, Thijssen DH, Low DA (2017). Reproducibility of four frequently used local heating protocols to assess cutaneous microvascular function. Microvasc Res.

[CR101] Rossi M, Cupisti A, Mariani S, Santoro G, Pentimone F (2002). Endothelium-dependent and endothelium-independent skin vasoreactivity in the elderly. Aging Clin Exp Res.

[CR102] Rossi M, Bazzichi L, Di Maria C, Franzoni F, Raimo K, Della Rossa A, Santoro G, Bombardieri S (2008). Blunted increase of digital skin vasomotion following acetylcholine and sodium nitroprusside iontophoresis in systemic sclerosis patients. Rheumatology (Oxford).

[CR103] Roustit M, Cracowski JL (2012). Non-invasive assessment of skin microvascular function in humans: an insight into methods. Microcirculation.

[CR104] Roustit M, Cracowski JL (2013). Assessment of endothelial and neurovascular function in human skin microcirculation. Trends Pharmacol Sci.

[CR105] Roustit M, Blaise S, Millet C, Cracowski JL (2010). Reproducibility and methodological issues of skin post-occlusive and thermal hyperemia assessed by single-point laser Doppler flowmetry. Microvasc Res.

[CR106] Rowell LB (1974). Human cardiovascular adjustments to exercise and thermal stress. Physiol Rev.

[CR107] Saumet JL, Kellogg DL, Taylor WF, Johnson JM (1988). Cutaneous laser-Doppler flowmetry: influence of underlying muscle blood flow. J Appl Physiol.

[CR108] Savage MV, Brengelmann GL, Buchan AM, Freund PR (1990). Cystic fibrosis, vasoactive intestinal polypeptide, and active cutaneous vasodilation. J Appl Physiol.

[CR109] Schulze E, Witt M, Fink T, Hofer A, Funk RH (1997). Immunohistochemical detection of human skin nerve fibers. Acta Histochem.

[CR110] Shibasaki M, Wilson TE, Cui J, Crandall CG (2002). Acetylcholine released from cholinergic nerves contributes to cutaneous vasodilation during heat stress. J Appl Physiol.

[CR111] Shibasaki M, Davis SL, Cui J, Low DA, DM MK, Durand S, Crandall CG (2006). Neurally mediated vasoconstriction is capable of decreasing skin blood flow during orthostasis in the heat stressed human. J Physiol.

[CR112] Smith CJ, Kenney WL, Alexander LM (2013). Regional relation between skin blood flow and sweating to passive heating and local administration of acetylcholine in young, healthy humans. Am J Physiol Regul Integr Comp Physiol.

[CR113] Smith CJ, Craighead DH, Alexander LM (2017). Effects of vehicle microdialysis solutions on cutaneous vascular responses to local heating. J Appl Physiol.

[CR139] Smith KJ, Argarini R, Carter HH, Quirk BC, Haynes A, Naylor LH, McKirdy H, Kirk RW, McLaughlin RA, Green DJ (2019). Novel noninvasive assessment of microvascular structure and function in humans. Med Sci Sports Exerc.

[CR114] Soderstrom T, Stefanovska A, Veber M, Svensson H (2003). Involvement of sympathetic nerve activity in skin blood flow oscillations in humans. Am J Physiol Heart Circ Physiol.

[CR115] Somlyo AV (2007). Cyclic GMP regulation of myosin phosphatase: a new piece for the puzzle?. Circ Res.

[CR116] Stefanovska A, Bracic M, Kvernmo HD (1999). Wavelet analysis of oscillations in the peripheral blood circulation measured by laser Doppler technique. IEEE Trans Biomed Eng.

[CR117] Stephens DP, Aoki K, Kosiba WA, Johnson JM (2001). Nonnoradrenergic mechanism of reflex cutaneous vasoconstriction in men. Am J Physiol Heart Circ Physiol.

[CR118] Stephens DP, Bennett LA, Aoki K, Kosiba WA, Charkoudian N, Johnson JM (2002). Sympathetic nonnoradrenergic cutaneous vasoconstriction in women is associated with reproductive hormone status. Am J Physiol Heart Circ Physiol.

[CR119] Stephens DP, Saad AR, Bennett LA, Kosiba WA, Johnson JM (2004). Neuropeptide Y antagonism reduces reflex cutaneous vasoconstriction in humans. Am J Physiol Heart Circ Physiol.

[CR120] Tartas M, Bouye P, Koitka A, Jaquinandi V, Tan L, Saumet JL, Abraham P (2005). Cathodal current-induced vasodilation to single application and the amplified response to repeated application in humans rely on aspirin-sensitive mechanisms. J Appl Physiol.

[CR121] Taylor WF, Johnson JM, O’Leary D, Park MK (1984). Effect of high local temperature on reflex cutaneous vasodilation. J Appl Physiol.

[CR122] Taylor NA, Machado-Moreira CA, van den Heuvel AM, Caldwell JN (2014). Hands and feet: physiological insulators, radiators and evaporators. Eur J Appl Physiol.

[CR123] Tew GA, Ruddock AD, Saxton JM (2010). Skin blood flow differentially affects near-infrared spectroscopy-derived measures of muscle oxygen saturation and blood volume at rest and during dynamic leg exercise. Eur J Appl Physiol.

[CR124] Tew GA, Klonizakis M, Crank H, Briers JD, Hodges GJ (2011). Comparison of laser speckle contrast imaging with laser Doppler for assessing microvascular function. Microvasc Res.

[CR125] Thompson-Torgerson CS, Holowatz LA, Flavahan NA, Kenney WL (2007). Cold-induced cutaneous vasoconstriction is mediated by Rho kinase in vivo in human skin. Am J Physiol Heart Circ Physiol.

[CR126] Thompson-Torgerson CS, Holowatz LA, Kenney WL (2008). Altered mechanisms of thermoregulatory vasoconstriction in aged human skin. Exerc Sport Sci Rev.

[CR127] Ungerstedt U, Hallstrom A (1987). In vivo microdialysis—a new approach to the analysis of neurotransmitters in the brain. Life Sci.

[CR128] Wallengren J (1997). Vasoactive peptides in the skin. J Invest Dermatol Symp Proc.

[CR129] Wallengren J, Ekman R, Sundler F (1987). Occurrence and distribution of neuropeptides in the human skin. An immunocytochemical and immunochemical study on normal skin and blister fluid from inflamed skin. Acta Derm Venereol.

[CR130] Wardell K, Braverman IM, Silverman DG, Nilsson GE (1994). Spatial heterogeneity in normal skin perfusion recorded with laser Doppler imaging and flowmetry. Microvasc Res.

[CR131] Whitney RJ (1949). The measurement of changes in human limb-volume by means of a mercury-in rubber strain gauge. J Physiol.

[CR132] Whitney RJ (1953). The measurement of volume changes in human limbs. J Physiol.

[CR133] Wilkins BW, Wong BJ, Tublitz NJ, McCord GR, Minson CT (2005). Vasoactive intestinal peptide fragment VIP10-28 and active vasodilation in human skin. J Appl Physiol.

[CR134] Wong BJ, Minson CT (2006). Neurokinin-1 receptor desensitisation attenuates cutaneous active vasodilatation in humans. J Physiol.

[CR135] Wong BJ, Minson CT (2011). Altered thermal hyperaemia in human skin by prior desensitization of neurokinin-1 receptors. Exp Physiol.

[CR136] Wong BJ, Wilkins BW, Minson CT (2004). H1 but not H2 histamine receptor activation contributes to the rise in skin blood flow during whole body heating in humans. J Physiol.

[CR137] Wong BJ, Tublitz NJ, Minson CT (2005). Neurokinin-1 receptor desensitization to consecutive microdialysis infusions of substance P in human skin. J Physiol.

[CR138] Zhao JL, Pergola PE, Roman LJ, Kellogg DL (2004). Bioactive nitric oxide concentration does not increase during reactive hyperemia in human skin. J Appl Physiol.

